# The role of the amygdala in the pathophysiology of panic disorder: evidence from neuroimaging studies

**DOI:** 10.1186/2045-5380-2-20

**Published:** 2012-11-20

**Authors:** Jieun E Kim, Stephen R Dager, In Kyoon Lyoo

**Affiliations:** 1 Department of Brain & Cognitive Sciences, Graduate School, Ewha Womans University, 52 Ewhayeodae-gil, Seodaemun-gu, 120-750, Seoul, South Korea; 2 Department of Radiology, School of Medicine, University of Washington, 1100 NE 45th St, Ste 555, WA 98105, Seattle, USA; 3 Division of Life and Pharmaceutical Sciences and Ewha Brain Institute, Ewha Womans University, 52 Ewhayeodae-gil, Seodaemun-gu, 120-750, Seoul, South Korea

**Keywords:** Panic disorder, Panic attack, Amygdala, Neuroimaging

## Abstract

Although the neurobiological mechanisms underlying panic disorder (PD) are not yet clearly understood, increasing amount of evidence from animal and human studies suggests that the amygdala, which plays a pivotal role in neural network of fear and anxiety, has an important role in the pathogenesis of PD. This article aims to (1) review the findings of structural, chemical, and functional neuroimaging studies on PD, (2) relate the amygdala to panic attacks and PD development, (3) discuss the possible causes of amygdalar abnormalities in PD, (4) and suggest directions for future research.

## Review

### Introduction

Panic disorder (PD) is characterized by repeated panic attacks, often accompanied with anticipatory anxiety, fear of losing control or sanity, or behavioral changes related to the attacks [1]. An epidemiological study conducted with a nationally representative sample estimated the lifetime prevalence of PD to be 4.5% [2]. A panic attack typically develops suddenly and reaches its peak within 10 minutes. Symptoms that accompany panic attacks include palpitations, chest pain, sweating, trembling, smothering, abdominal distress, dizziness, and fear of dying. It is estimated to be highly prevalent, with the percentage of people who experience a panic attack at least once in their lifetime reaching up to 28.3% [2-4]. Panic responses, when proximal predatory threat is approaching or is actually present, are adaptive in the sense that they prepare animals to fight vigorously or flee (“fight or flight response”) [5]. During panic attacks, however, an intense fear response to aroused sympathetic activity is manifested in the absence of actual danger [1]. Animal model studies, genetic studies, and human neuroendocrinology and neuroimaging studies have provided important insights [5-9], although the neurobiological underpinnings of the panic attack and PD are not yet completely understood [10].

Neuroendocrinological studies have implicated dysfunction of the hypothalamic-pituitary-adrenal axis, although this perturbation occurs only later in the course of the disorder, after the development of anticipatory anxiety and associated distresses [11,12]. Apart from higher levels of *COMT* Val158Met polymorphism in PD patients, the role of genes in PD susceptibility has not yet been defined [7].

Neuroanatomical correlates that may be responsible for PD pathogenesis have been suggested [6,13]. The implicated brain areas are the amygdala, thalamus, hypothalamus, and brain stem regions including the periaqueductal gray, parabrachial nucleus, and locus ceruleus [6,13]. In particular, the amygdala has been suggested to have a critical role in PD [14], while there have been a few studies that indicate otherwise [15].

The aim of this review is to describe and discuss the neuroimaging findings and current hypotheses on the role of amygdala in the pathophysiology of PD.

### The amygdala

The amygdala is known to have 13 nuclei, which can be categorized into lateral, basal and central subregions [16]. In humans, nuclei of the amygdala are usually grouped as laterobasal subgroup including both lateral and basal nuclei, centromedial subgroup, and cortical subgroup. These subgroups deal with fear [17], a process which can be schematically illustrated as follows: the lateral subgroup receives information from the cortical and subcortical areas, the basal subgroup inter-connects the lateral and central subgroups, and sends the output to the cortical areas, and the central subgroup conveys the information to the brain regions including hypothalamus and periaqueductal gray [17]. The laterobasal and central subgroups are also connected with bed nucleus of the stria terminalis, which also projects to hypothalamus, cerebellum, and brain stem areas [18].

**Figure 1:**
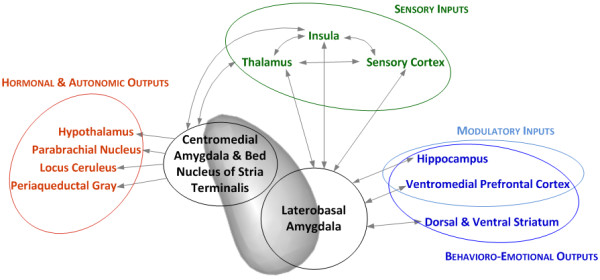
Schematic diagram of inputs to and outputs from the amygdala, relevant to panic disorder pathogenesis.

The Figure 1 shows the simplified inputs and outputs of the amygdala, which have been reported to be associated with PD pathogenesis [13,18]. Theoretically, disruption in any of these brain areas and connections along these areas, or any imbalance in the network can cause maladaptive and exaggerated fear responses such as panic attacks, increased basal anxiety or arousal [19], and excessive worrying [18,20]. Consistent with this postulation, structural, chemical, and functional alterations in these amygdalar areas have been reported in neuroimaging studies in patients with PD (Tables 1, 2, 3, 4, 5; Additional files 1, 2, 3). It has been suggested that the amygdala has a critical role in the development of panic attacks and the pathogenesis of PD [14,17,18].

**Table 1:** Structural neuroimaging findings in panic disorder [computerized tomography]

**Study**	**Subjects**	**No.of subjects (female)**	**Mean age (SD)**	**Clinical state**	**Duration of illness**	**Comorbid Agoraphobia**	**Medication status**	**Imaging method**	**Amygdala**	**Hippocampus**	**Ventromedial Prefrontal cortex**	**Other brain regions**
Wurthmann, 1998 [21]	PD	21 (16)	35.52 (5.95)	PAS 24.47 (8.25)	5.24 (5.37)	Current, n = 18	Medication naive, n = 10; BDZ, n = 4; BDZ and antidepressants, n = 2; BDZ and neuroleptics, n = 1; Beta blockers, n = 1; Antidepressants, n = 3	CT, 5-mm sections in posterior fossa, 10-mm sections in central hemispheres	Not assessed	Not assessed	Not assessed	CT abnormalities: Frontal CSF space▲
	HC	21 (16)	35.52 (6.68)									
Lepola, 1990 [22]	PD	31 (23)	37 (Range 16–52)		6.4 (Range 4–35)	With/Without -	At some period treated with psychotropic agents, n = 32	CT, axial, 4-mm-thick sections in posterior fossa and basal forebrain, 4-/8-mm sections in cerebral hemispheres, n = 30	Not assessed	Not assessed	Not assessed	CT abnormalities: Cerebral atrophy, n = 3; Lacunar infarcts, n = 2; Enlarged lateral ventricles, n = 1

Abbreviations: *BDZ*, benzodiazepine; *CSF*, cerebrospinal fluid; *HC*, healthy control; *PAS*, Panic and Agoraphobia Scale; *PD*, panic disorder; *SD*, standard deviation.

**Table 2:** Structural neuroimaging findings in panic disorder [diffusion tensor imaging]

**Study**	**Subjects**	**No.of subjects(female)**	**Mean age(SD)**	**Clinical state**	**Duration of illness(years)**	**Comorbid Depression**	**Comorbid Agoraphobia**	**Medication status**	**Field strength(Tesla)**	**Slice thickness(mm)**	**Amygdala**	**Hippocampus**	**Ventromedial Prefrontal cortex**	**Other brain regions**
Han, 2008 [23]	PD	24 (10)	31.9 (6.8)	PDSS 9.5 (6.4)	3.6 (1.9)	HDRS-17 scores of PD patients were not different from those of HC subjects.	Not mentioned	Combination of antidepressants and anxiolytics	3.0	3.5	Not assessed	Not assessed	Left anterior cingulate, right posterior cingulate FA value▲	Not assessed
	HC	24 (10)	30.6 (5.1)	-	-	-	-							

Abbreviations: *FA*, fractional anisotropy; *HC*, healthy control; *PD*, panic disorder; *PDSS*, Panic Disorder Severity Scale; *SD*, standard deviation.

**Table 3:** **Functional neuroimaging findings in panic disorder **[**near**-**infrared spectroscopy**]

**Study**	**Subjects**	**No.of subjects(female)**	**Mean age(SD)**	**Comorbid Depression**	**Comorbid Agora**-**phobia**	**Medication status**	**Study paradigm**		**Amygdala**	**Hippocampus**	**Ventromedial Prefrontal cortex**	**Other brain regions**
Tanii, 2009 [24]	PD	71 (52)	37.9				Genotyping (*COMT* Val158Met polymorphism)	Met/Met, n = 8	-	-	-	Right LPFC O_2_Hb▲ in Met/Met
								Val/Met, n = 29				
								Val/Val, n = 34				
Dresler, 2009 [25]	PD	1 (0) Case study	44	Current		Medicated with doxepine, zopiclone and amitriptyline	Emotional Stroop task with words (Neutral/panic-related), before/after repetitive transcranial magnetic stimulation (rTMS) treatment to left PFC		-	-	-	Neutral/panic-related: DLPFC O_2_Hb▲ after rTMS treatment (small superiority for Panic-Related)
Akiyoshi, 2003 [26]	PD	23 (12)	26.5 (7.6)	None	Current, n = 14	Alprazolam/paroxetine, n = 19	Presentation of visual stimuli (Neutral/anxiety-relevant/ anxiety-irrelevant but emotion-relevant)		-	-	-	Anxiety-relevant/ anxiety-irrelevant but emotion-relevant: left frontal O_2_Hb▼
	HC	31 (15)	24.1 (6.4)									

Abbreviations: *DLPFC*, dorsolateral prefrontal cortex; *HC*, healthy control; *LPFC*, lateral prefrontal cortex; *Met*, methionine; *O*_*2*_*Hb*, oxygenated hemoglobin; *PD*, panic disorder; *PFC*, prefrontal cortex; *SD*, standard deviation; *Val*, valine.

**Table 4:** **Functional neuroimaging findings in panic disorder **[**electroencephalography**]

** Study**	**Subjects**	**No.of subjects(female)**	**Mean age(SD)**	**Comorbid Depression**	**Comorbid Agora**-**phobia**	**Medication status**	**Findings**
Eser, 2009 [27]	HC (CCK-4 injection)	77 (0)		None	None	None taking medication	No correlations between CCK-4 induced panic symptom severity and loudness dependency of auditory evoked potentials
Lepola, 1990 [22]	PD	31 (23)	37 (Range 16–52)			At some period treated with psychotropic agents, n = 32	Slow-wave activity▲ in 13 patients.
Stein, 1989 [28]	PD	35 (24)	35 (8)			n = 33 free of all psychotropic drugs for a minumum of 2 weeks. n = 2 has taken a low dose of a benzodiazepine 2 days before evaluation.	n = 5 (14%) had nonspecific abnormal EEGs. None had evidence supportive of an ictal process.

Abbreviations: *CCK-4*, cholecystokinin tetrapeptide; *EEG*, electroencephalography; *PD*, panic disorder.

**Table 5:** **Chemical neuroimaging findings in panic disorder **[**magnetic resonance spectroscopy**]

**Study**	**Subjects**	**No.of subjects(female)**	**Mean age(SD)**	**Clinical state**	**Comorbid Depression**	**Comorbid Agora**-**phobia**	**Medication status**	**Field strength(Tesla)**	**Imaging method**	**Study paradigm**	**Amygdala**	**Hippocampus**	**Ventromedial Prefrontal cortex**	**Other brain regions**
**Induced Panic Attack**														
Friedman, 2006 [29]	PD	9 (4)	42.7 (15.3)	API 7.39 (7.19) Anxiety ratings 1.56 (1.33) Panic ratings 2.33 (1.94)	None		Fluoxetine, n = 8; Gabapentin, n = 1	1.5	^31^P-MRS	Regulated hyperventilation				Blunted pH response in PD
	HC	11 (8)	31.5 (9.6)	API 1.63 (2.06) Anxiety ratings 0.09 (0.30) Panic ratings 0 (0)										
Layton, 2001 [30]	PD	6 (3)		API 20.3 (5.2) Panic severity 6.7 (2.4) Anxiety severity 2.8 (1.5)				1.5	PEPSI	Panic induced by initial intravenous sodium lactate infusion/second infusion with gabapentin responders, n = 4; placebo responders, n = 2				No significant difference in Lactate/NAA levels between conditions (although gabapentin reduced clinical response to infusion)
Dager, 1999 [31]	PD	15 (8)	33.1 (11.7)	API 8.9 (8.2) Panic Severity 2.4 (3.0) Anxiety Severity 2.1 (1.3)	None		Free for 1 month	1.5	PEPSI	Panic induced by intravenous sodium lactate infusion (Panickers, n = 12)				Panickers: Earlier global Lactate/NAA▲
	HC	10 (6)	32.7 (7.4)	API 1.5 (1.8) Panic Severity 0.2 (0.4) Anxiety Severity 0.5 (0.9)										
Dager, 1997 [32]	PD	13 (8)	36.2 (9.1)	API 25.7 (9.7) Panic Severity 8.0 (1.9)	None		Free for 1 month	1.5	^1^ H-MRS	Panic induced by intravenous sodium lactate infusion (Panickers, n = 10)				PD patients: Insular cortex Lactate/NAA▲
	HC	10 (6)	37.7 (7.5)	API 4.5 (2.5)										
Dager, 1994 [33]	PD	8 (4)	38.6 (7.2)		None		Fluoxetine, n = 3; Fluoxetine + alprazolam, n = 1; Imipramine, n = 1	1.5	^1^ H-MRS	Panic induced by intravenous sodium lactate infusion (Panickers, n = 3)				Panickers: Insular cortex Lactate/NAA▲
	HC	8 (2)	36.0 (6.8)											
**Others**														
Trzesniak, 2010 [34]	PD	25 (19)	39.2 (9.9)		History, n = 13	Current, n = 10	SSRI, n = 8 Clorimipramine, n = 2 BZD, n = 3 BZD + SSRI, n = 3	1.5	^1^ H-MRS	-		Left hippocampus NAA/Cr▼		
	HC													
Hasler, 2009 [35]	PD	17 (12)	34.2 (10.1)	PD onset 22 (8.9) yrs PD duration 21 (31) mts PDSS 7.5 (3.0); PSS 18 (5.4) HAM-A 9.6 (6.2) HAM-D 9.6 (7.4)	Current MDD, n = 7	Current, n = 7	Medication naive, n = 9; Free for 3 months, n = 8	3.0	^1^ H-MRS	-			No significant difference GABA, Glx, CHO, NAA	No significant difference in DM/DALPFC GABA, Glx, CHO, NAA
	HC	17 (12)	35.1 (11.8)											
Maddock, 2009 [36]	PD	15 (10)	37.5 (9.2)	STAI-S 41.3 (10.6) API 2.1 (2.7) PAS 22.7 (10.5) ASI 36.9 (14)	Past MDD, n = 1	Current, n = 14	Untreated, n = 15	1.5	^1^ H-MRS	Vigilance task with visual stimuli				Visual cortex Lactate/NAA▲
	HC	15 (10)	37.1 (6.9)	STAI-S 25.4 (9.8) API 0.3 (1.0)										
Ham, 2007 [37]	PD	24 (10)	31.9 (6.8)	PDSS 9.5 (6.4) HAM-D 4.7 (5.0)	Not current		Combination of antidepressants and anxiolytics	3.0	^1^ H-MRS				GABA▼ Lactate▲ Cho▲	Basal ganglia GABA▼
	HC	24 (10)	30.5 (5.2)	HAM-D 5.2 (4.1)										
Goddard, 2004 [38]	PD	10 (5)	36 (11)		None	With/Without	Medication naive, n = 5; Free for 3 months, n = 2; BDZ until 1 week before, n = 3	2.1	^1^ H-MRS	Acute oral, open-label BDZ (clonazepam) administration				Occipital cortex GABA▼
	HC	10 (5)	31.5 (9.6)											
Massana, 2002 [39]	PD	11 (6)	34.27 (6.92)		None	All with some degree	All were antidepressant-naive and medication-free for 2 weeks	1.5	^1^ H-MRS	-			No significant difference in medial frontal cortex	Right medial temporal lobe Cr + PCr▼ ;
	HC	11 (6)	34.55 (6.95)											
Goddard, 2001 [40]	PD	14 (8)	37 (10)	PDSS 13 (4), n = 13HAM-A 17 (8) 25-item HAM-D 20 (10) 17-item HAM-D 14 (6)CRAS 31 (16)	None	With/Without	Medication naive, n = 9; Free for 3 months, n = 2; BDZ until 1 week before, n = 3	2.1	^1^ H-MRS	-				Occipital cortex GABA▼ , significant in Female Panickers
	HC	14 (8)	31.5 (9.6)											
Shioiri, 1996 [41]	PD	18 (10)	35.9 (9.1)	HAM-A 9.8 (6.3)	None		All on BDZ	1.5	^31^P-MRS	-				Frontal lobe slight Pi▼ ; Frontal lobe PCr L > R asymmetry
	HC	18 (10)	35.3 (11.4)											

Abbreviations: *API*, Acute Panic Inventory; *ASI*, Anxiety Sensitivity Index; *BDZ*, benzodiazepine; *Cho*, choline; *Cr*, creatine; *CRAS*, Clinician-Rated Anxiety Scale; *DM/DALPFC*, dorsomedial/dorsal anteromedial prefrontal cortex; *GABA*, gamma-aminobutyric acid; *Glx*, glutamte + glutamine; *HAM-A*, Hamilton Anxiety Rating Scale; *HAM-D*, Hamilton Depression Rating Scale; *HC*, healthy control; *NAA*, N-acetyl aspartate; *PAS*, Panic and Agoraphobia Scale; *PCr*, phosphocreatine; *PD*, panic disorder; *PDSS*, Panic Disorder Severity scale; *PEPSI*, proton echo-planar spectroscopic imaging; *Pi*, inorganic phosphate; *SD*, standard deviation; *SSRI*, selective serotonin reuptake inhibitor; *STAI-S*, State Trait Anxiety Index-State version.

### Structural abnormalities of the amygdala in patients with PD

There have been only a handful of structural neuroimaging studies that examined neuroanatomical alterations in patients with PD (Tables 1, 2, Additional file 1), relative to the number of studies in patients with other anxiety disorders such as post-traumatic stress disorder and obsessive compulsive disorder [42-45].

In earlier studies using the computerized tomography (Table 1), it may have not been possible to evaluate amygdalar structural alterations due to insufficient spatial resolution and tissue contrast.

All magnetic resonance imaging studies in which amygdalae were manually traced have consistently reported amygdalar volume reduction in patients with PD [46-48] (Additional file 1). In the report of Uchida and colleagues, there was a trend-level significance for bilateral amygdalar volume reduction [48], while two other studies reported a statistically significant bilateral amygdalar volume reduction [46,47]. In addition to the relatively small sample size, the fact that amygdalae were traced on 2 mm-thick reformatted magnetic resonance (MR) images in the study of Uchida and colleagues [48] may have resulted in a comparatively greater error range, which could have undermined the power of detecting volume differences of small structures. In the report of Massana and colleagues, 1.2 mm-thick isocubically reformatted MR images were used for tracing [47]. Although the voxel size of images used for tracing is not described in the report of Hayano and colleagues [46], it is likely that 1.5-mm thick native images were used according to the standard protocol of manual segmentation in 3D slicer (http://www.slicer.org) which was the image analysis software adopted in the study.

Among five studies that used whole brain-wise approach of voxel-based morphometry (VBM) (Additional file 1), the report of Asami and colleagues noted amygdalar volume reduction [49]. It has been suggested that VBM approach may have limited sensitivity for identifying *a priori*-specified structural differences unless analytical approaches such as small volume correction are applied. This indicates that a large sample size may be required for the reliable results in VBM studies [50-52]. Thus, it is not surprising that the study of Asami et al., which had the positive finding in the amygdala, had the largest sample size among these five VBM studies.

The effect size for group differences was greater in the right amygdala than the left amygdala across all four studies that have reported the amygdalar volume reduction [46-49]. In the study of Asami et al., a statistically significant amygdalar volume reduction was noted only in the right hemisphere. For the studies of Hayano et al. and Uchida et al., Ray and Shadish’s equation 2 [53] was used to compute Cohen’s *d* values based on *t* score and sample sizes for each group presented in the paper (Cohen’s *d* = 0.77 for the right amygdala; 0.60 for the left amygdala in Hayano et al.; Cohen’s d = 0.78 for the right amygdala [8% volume differences]; 0.76 for the left amygdala [5% volume differences] in Uchida et al.). Regarding the report of Massana et al., Cohen’s *d* for amygdalar volume differences was 2.07 for the right amygdala and 1.66 for the left amygdala.

Reasons for the potentially greater deficits in the right amygdala than in the left amygdala in patients with PD may be understood in a large body of literature on hemispheric organization for processing emotions such as fear. The right hemisphere has long been considered to have dominance over the left hemisphere regarding emotional behaviors [54,55]. Theories that suggest the lateralized role of right and left hemispheres for different aspects of emotions have arisen [56]. Sackeim and colleagues proposed that the right hemisphere may have dominance especially in processing negative emotions [57]. Regarding fear processing and the amygdala, more recent neuroimaging studies indicated that the right amygdala is primarily involved in processing acquired fear, while the left amygdala is particularly involved in processing innate fear [58]. This lateralization has been interpreted in the context that innate fear may require more conscious and linguistic processing of the stimuli. On the other hand, acquired fear may not require as much conscious elaborations of the stimuli since the response would rather be automatic [58]. In PD, enhanced conditionality of fear (i.e., a tendency to acquire fear more easily) with resistance to extinction has been considered as one of the core elements of the pathology [59,60].

Because of the cross-sectional nature of the studies, it is not appropriate to infer the causality of the relationship between PD and reduced amygdalar volume. However, although speculative, some of the evidence indicates that amygdalar deficits, particularly the deficit in the right amygdala, may predispose to PD. Massana and colleagues found that amygdalar volume reduction was noted in both subgroups of PD patients with long duration of illness (>6 months) and those with relatively short duration of illness (<6 months). There were no associations between the magnitude of amygdalar volume reduction and clinical measures including panic symptom severity and illness duration [49]. In Uchida et al., correlational results between amgydalar volume and clinical measures were not reported [48]. In the study of Hayano et al., the right amygdalar volume of patients with PD had a negative correlation with the neuroticism score of NEO personality inventory-revised, the measure of enduring tendency toward experiencing negative emotional states, while the left amygdalar volume showed a negative correlation with the state anxiety score from the State-Trait Anxiety Inventory, the measure of anxiety severity [46]. This conjecture is entirely speculative and subsequently urges further studies.

It is less likely that amygdalar deficits in patients with PD are due to the use of antidepressants. The total daily dose of antidepressant was not associated with the amygdalar volume reduction [49]. The study of Massana and colleagues was conducted in patients who are antidepressant-naive [47]. Also, a meta-analysis in patients with major depressive disorder has suggested that antidepressant medication increases the amygdalar volume, rather than causing amygdalar deficits [61].

Since the amygdala is composed of many different subnuclei with distinctive connections and functions [62], researchers are interested in whether there would be subregional specificity of the amygdalar deficit. Hayano and colleagues investigated which subregion of the amygdala might show more deficits in patients with PD [46]. They employed the optimized voxel-based morphometry with small volume correction using bilateral amygdalar masks. Volume deficits were noted in the amygdalar subregion that might correspond to the corticomedial subregional group. Central and medial subnuclei that are parts of the corticomedial subregion have been implicated for autonomic responses to fear stimuli [63]. However, the laterobasal subregion of the amygdala has also been reported to be involved in the pathogenesis of PD [64]. Further studies that adopt strategies that may allow finer registration among inter-subject amygdalae [65-67] may be needed to confirm the subregional findings.

Evaluating the structural connectivity among the amygdala and other brain regions on diffusion tensor images would provide important additional information [68]. Relative to healthy comparison subjects, patients with PD had a greater fractional anisotropy value in the left anterior and right posterior cingulate regions [23] (Table 1). These regions may have a role in maintaining visceromotor homeostasis through its interconnections with the amygdala [69]. Recently, with the use of high quality diffusion tensor images and T1-weighted images, evaluating the connections among amygdalar subregions and other brain regions has become possible [70,71]. Studies with this approach would also enhance the understanding on the roles of amygdalar subregions.

### Functional abnormalities of the amygdala in patients with PD

Functional neuroimaging techniques that have been used for studying PD include functional magnetic resonance imaging (fMRI), positron emission tomography (PET), single photon emission computed tomography (SPECT), electroencephalography (EEG), and near-infrared spectroscopy (NIRS). Among these, NIRS and EEG may not be suitable for assessing amygdalar activity due to a relatively superficial penetration depth [72] (Tables 2 and 4).

There have been PET and SPECT studies that used neutral state paradigms. Earlier studies with a region-of-interest approach did not find significantly different amygdalar activity [73-75] (Additional file 2). As De Cristofaro and colleagues commented [74], image resolutions of SPECT and PET may not be sufficient for delineating the amygdala from other adjacent structures. A neutral-state PET study which used a whole brain-wise approach [76] also did not find amygdalar metabolic differences, while in a more recent study [77], a greater metabolism in bilateral amygdalar regions was noted in patients with PD compared with healthy comparison subjects. The authors conjectured that this discrepancy may have stemmed from differences in image resolution, sample size, and subjects’ age [77].

Among three fMRI studies that captured brain regional activation during spontaneous panic attacks, two studies conducted in patients with PD or specific phobia have found increased right amygdalar activation [78,79]. A study conducted in an individual with restless leg syndrome but without any history of psychiatric disorders has noted the relationship of the left amygdalar activity and the heart rate [80] (Additional file 3). A PET study could not find the difference of the amygdalar blood flow in a healthy volunteer who experienced a panic attack during fear conditioning in comparison with 5 healthy volunteers who did not experience panic attacks during the same fear conditioning sessions [81].

Symptom provocation paradigms were used both in healthy volunteers and in patients with PD (Additional file 2, 3). When challenged with cholecystokinin tetrapeptide (CCK-4) or procaine, healthy volunteers showed increased amygdalar activation [82] or increased regional cerebral blood flow [83-85]. In patients with PD, CCK-4 or doxapram intravenous injection did not elicit significantly greater amygdalar activation [86,87]. Due to relatively modest sample sizes, there is a risk for the type II error, precluding the possibility of drawing any conclusions from these negative findings. In a report of Boshuisen et al., PD patients experiencing anticipatory anxiety showed significantly decreased regional cerebral blood flow in the right amygdala [88]. The authors commented on the possibility that cortical inhibitory effects in response to the intense anticipatory anxiety may have depressed the amygdalar activity. It has also been suggested that this might reflect the functional impairment of the amygdala to cope with the anticipatory anxiety [88].

Cognitive activation probes to evaluate amygdalar function have been used in functional neuroimaging studies of PD [9]. In most studies which used fMRI, patients with PD showed altered amygdalar activation level (Additional file 3) except a study with motor activation paradigms [89] and a few with a relatively small sample size [90,91]. In addition, Maddock et al., suggested that repeated presentation of the threat-related stimuli in their study (20 words being repeated eight times) may have habituated the amygdalar response [90]. This may have resulted in the negative finding in this region.

Among studies that report altered amygdalar activation in PD patient group relative to comparison group, both higher and lower amygdalar activation level was found [92]. While most of the studies reported increased amygdalar activation, two studies [93,94] noted deactivated the amygdala in patients with PD relative to healthy comparison subjects. Statistical activation maps of comparison between the threat condition vs. the safe condition in the study of Tuescher and colleagues showed less activation in the amygdala of PD patients in the threat condition relative to post traumatic stress disorder patients [93]. However, this was mainly due to amygdalar hyperactivation in PD patients in the safe condition relative to threat condition, while post-traumatic stress disorder patients showed increased amygdalar activation in the threat condition. The fact that patients with PD were compared to those with post-traumatic stress disorder in this study, and the difference in the experimental paradigm from other studies precludes direct comparison of this study result with those from other studies [95]. In this study, patients were instructed that visual presentation of the square with a certain color would mean that an electrodermal stimulation can occur at any time. Actual stimulation did not happen during scanning. This may have caused anticipatory anxiety as in the study of Boshuisen and colleagues [88], in which patients with PD also exhibited decreased regional cerebral blood flow in the amygdala. Deactivation of the amygdala in response to presentation of fearful faces [94] may in part be due to the facts that most of the patients were taking antidepressants [92], since antidepressants have been reported to decrease the amygdalar activity [96]. In addition, it should be considered that the relatively lenient statistical threshold (p < 0.05) for *a priori*-hypothesized regions of the anterior cingulate cortex and the amygdala [94]. It is possible that only certain subgroups of patients with PD exhibit amygdalar hyperactivation [92]. Only women were responsive to fearful faces [97]. Patients with the *COMT* 158 val allele showed hyperactivation of the amygdala in response to fearful faces [98]. In a relatively small sample size, the heterogeneity with regard to genetic polymorphism and sex may make it hard to detect the increased amygdalar activation, if any [99]. Baseline anxiety and associated physiologic changes may also confound functional neuroimaging findings [19].

Findings regarding laterality of amygdalar activation (Additional file 3) are less consistent than those of amygdalar volume (Additional file 1). Left and right amygdalae are known to be involved in different aspects of fear processing [58]. Direct comparison of results is not appropriate since the experimental paradigms are slightly different with each other [95]. In studies with a relatively small sample size, differences in the number of subjects with right or left-handedness may confound the laterality findings [95].

There have been reports that investigated amygdalar function in relation to the treatment (Additional file 2). When untreated patients and treated patients were compared with healthy comparison subjects, patients treated with antidepressants showed no difference in amygdalar serotonin binding potential, while untreated patients showed significantly lower serotonin binding potential [100]. Treatment with paroxetine for 12 weeks in psychotropic medication-naive PD patients altered amygdalar glucose metabolism [101]. In the report of Prasko and colleagues, treatment with antidepressants did not change the amygdalar glucose metabolism [102]. This may have stemmed from exposure to antidepressants prior to study participation [102]. Cognitive behavioral therapy did not change the amygdalar glucose metabolism [102,103]. The number of studies in regards to treatment-related changes in amygdalar function is small and more studies are required for conclusions.

### Potential mechanisms underlying altered amygdalar function and structure in patients with PD

Structural and functional neuroimaging findings with regard to the amygdala in patients with PD are not perfectly consistent, but a pattern suggested by one of the neurocircuitry models may be noted [13]. Increased reactivity of the amygdala with structural deficits, similar to the findings in other anxiety disorders such as post-traumatic stress disorder [92]. Whether structural deficits of the amygdala cause its hyper-reactivity or hyperactivation of the amygdala for a prolonged period elicits overuse atrophy is not known [104].

Brain physiological processes underlying altered amygdalar function and structure may be partly expounded by the findings from molecular imaging and chemical neuroimaging studies (Additional file 2, Table 5). Decreased gamma amino-butyric acid (GABA)-benzodiazepine receptor binding has been reported in the medial temporal lobes that may include amygdalar regions [105,106] (Additional file 2). In the amygdala, decreased 5-HT1_A_ receptor binding has also been reported [100]. Both of these receptors are involved in inhibitory neurotransmission. Defective inhibition of the amygdalar activity may result in paroxysmal elevations in anxiety [105]. Chemical neuroimaging studies have reported lower levels of GABA in patients with PD than healthy comparison subjects [37,40], which were not reversed with acute benzodiazepine challenge [38]. Decreases in the GABA level may cause dysfunction in GABAergic inhibition of brain activity [107]. Morever, experimentally lowered GABA level caused panic-like behaviors in rats [108].

Findings from chemical neuroimaging studies suggest metabolic disturbances in the brain of patients with PD [19] (Table 5). Hypermetabolic state was suspected based on the findings that show depletion of phosphocreatine and creatine [92,109] in patients with PD, consistent with functional neuroimaging findings. Shioiri and colleagues reported higher level of inorganic phosphate in patients with PD [41]. Inorganic phosphate is known to be accumulated when creatine phosphate is broken down during anaerobic metabolism [110]. A rapid rise of the brain lactate level in response to physiological challenge (vigilance task with visual stimuli) also suggests an intrinsic metabolic disturbance [19,36].

Genetic polymorphism and early-life experiences may also contribute to amygdalar abnormalities. Specially, the *COMT* Val158Met polymorphism that affects the amygdalar structure, function, and receptor expression [98,111-113] has also been reported to be associated with PD development [7].

Data from the National Comorbidity Survey showed a positive association between early-life traumatic experiences and later development of PD [114]. PD patients who experienced early-life traumatic events had more severe symptom profile [115]. Relationships between early-life traumatic experiences and amygdalar dysfunction have also been reported [116].

### The role of amygdalar pathology in developing PD

Electrical or chemical stimulation of the amygdalar central nucleus causes constellation of symptoms that are very similar to those of panic attacks [63]. Electrolytic lesions made in the central nucleus would also disrupt fibers connecting laterobasal nucleus and bed nucleus of the stria terminalis, which has outputs to the hypothalamus and brain stem [18], where the centers for autonomic and neuroendocrine response regulations are located. When the efferent fibers from the central nucleus are stimulated, similar effects are produced [117]. By abruptly blocking the tonic GABAergic inhibition in the laterobasal subregion of the amygdala, symptoms mimicking panic attacks are induced [64]. These animal studies suggest the role of the amygdala in the pathogenesis of PD.

Earlier models to explain the neurobiology of PD have underscored the hypersensitivity to carbon dioxide, namely “false suffocation alarm theory [118,119],” although it has been revised since [120]. The following neurocircuitry model [13] is well suited for explaining the role of the ‘hyperactive and smaller’ amygdala [104] in the pathogenesis of PD for the fairly consistent findings.

Findings of Ziemann et al., suggest the role of the amygdala in sensing central acidosis [121] which has been considered as one of the core pathophysiological processes in PD [122]. In chemical neuroimaging studies in which neurometabolite changes were measured during induced panic attacks [31-33,102], lactate/n-acetyl aspartate level increased during panic attacks (Table 5). Hyperventilation or sodium lactate infusion leads to a metabolic alkalosis, which presumably augments neuronal activity [123] and alters the redox state to glycolytic metabolism producing lactate [29,31]. The report of Shioiri and colleagues which found increased level of inorganic phosphate, a potential indicator of anaerobic metabolism, indirectly supports the possibility of the redox shift to glycolysis [41]. It is also possible that lactate builds up presumably due to reduced cerebral blood flow [124]. Elevated brain lactate level, which would be associated with decreased brain pH or exaggerated alkalotic buffering [29], may trigger a panic attack by stimulating the amygdala through the chemosensing ion channel [121].

### The role of the amygdala in progression to PD

Panic attacks do not necessarily progress to PD [3]. Bouton and colleagues [60] proposed that among all individuals who experience panic attacks, only those who have increased conditionality would develop PD. As panic attacks recur, the association would become even stronger [125,126]. Consistent with this hypothesis, enhanced conditionality [127] and resistance to extinction in patients with PD have been reported. Altered anxiety neurocircuitry including the amygdala in PD may in part be responsible for this enhanced conditionality and resistance to extinction.

The amygdala projects to the striatum and cortical areas, and induce behavioral changes [62]. Phobic avoidance or agoraphobia, often associated with PD, may reflect the amygdala’s influence on these areas [128]. Unlike the general belief that ’cognition rules over emotion,’ there is evidence that emotion modulates cognition from perception and attention [17] to higher domains of judgment and reasoning [129]. Irrational worry about the connotation of the attacks, one of the PD core symptoms, may also be partly attributed to the amygdalar structural and functional abnormalities.

### Summary and recommendations

The amygdala has been reported to have a crucial role in the pathophysiology of PD. In animal studies, behaviors similar to those of panic attacks were observed when the amygdala was stimulated. Increased amygdalar activity with volumetric deficits was noted in patients with PD, although not always consistent. This altered function and structure of the amygdala may be partly due to dysregulated brain metabolism [31-33,121]. As potential causes of amygdalar abnormality in PD, *COMT* Val158Met polymorphism and early life traumatic experiences can also be suggested [112,130,131].

Enhanced conditionality and resistance to extinction are known as risk factors in progression from panic attack to PD. As an important element of the anxiety neurocircuitry, altered amygdalar function and structure may facilitate the progression to PD. Typical PD symptoms such as phobic avoidance and irrational worry of panic attacks may also be attributed to amygdalar structural and functional abnormalities.

Partly inconsistent findings from human neuroimaging studies are possibly due to sample heterogeneity, small sample size, and limitations of neuroimaging techniques. More studies with a larger sample size are warranted. In terms of technical difficulties, subregional analysis of the amygdala, which detects alterations in subregions of the amygdala separately, could be a novel strategy for future research, since each nucleus might have a distinct role in the pathophysiology of PD. This approach has already shown its potential in structural neuroimaging studies [65,66] and functional neuroimaging studies, examining neural circuitry in healthy individuals [132,133]. A postmortem study, investigating association between pathology in amygdalar subdivisions of patients with Parkinson’s disease [134] and premortem anxiety symptoms, provides another insight for a new strategy.

## Conclusions

The amygdala, the hub of fear processing networks, is closely associated with the pathogenesis of PD as well as panic attack. Further studies in well-defined larger samples, with more sophisticated research designs and advanced technologies would promise a better understanding on the role of the amygdala in the pathophysiology of PD.

## Abbreviations

PD: Panic disorder;MR: Magnetic resonance;VBM: Voxel-based morphometry;fMRI: Functional magnetic resonance imaging;PET: Positron emission tomography;SPECT: Single photon emission computed tomography;EEG: Electroencephalography;NIRS: near-infrared spectroscopy;CCK-4: cholecystokinin tetrapeptide;GABA: gamma amino-butyric acid

## Competing interests

IKL has received research support from AstraZeneca, Lundbeck, and GSK. All other authors declare that they have no competing interests.

## Authors’ contributions

JEK, SRD and IKL have contributed to conception and design of the review. JEK drafted the manuscript. All authors have done a critical revision of the manuscript and approved the final version of the manuscript.

## Authors’ information

JEK is an assistant professor at the Department of Brain & Cognitive Sciences, Ewha Womans University Graduate School, Seoul, South Korea. SRD and IKL are professors at the Department of Radiology, University of Washington School of Medicine, Seattle, USA. IKL is a professor at the Division of Life and Pharmaceutical Sciences, Ewha Womans University College of Pharmacy and a director of Ewha Brain Institute, Ewha Womans University, Seoul, South Korea.

## Supplementary Material

Additional file 1 **Structural Neuroimaging Findings in Panic Disorder [Magnetic Resonance Imaging] [**46-49,135-142**].** Click here for file

Additional file 2 **Functional Neuroimaging Findings in Panic Disorder [Positron Emission Tomography/Single Photon Emission Computed Tomography] [**73-77,81,83-86,88,100-103,105,106,143-148**].** Click here for file

Additional file 3 **Functional Neuroimaging Findings in Panic Disorder [Functional Magnetic Resonance Imaging] [**78-80,82,87,89-91,93,94,97-99,149-153**].** Click here for file
