# Supplementary material for: The role of the amygdala in the pathophysiology of panic disorder: evidence from neuroimaging studies
Source: Biol Mood Anxiety Disord. 2012 Nov 20;2:20. doi: 10.1186/2045-5380-2-20 (PMC3598964; doi:10.1186/2045-5380-2-20)
Supplement: Additional file 1 — Structural Neuroimaging Findings in Panic Disorder [Magnetic Resonance Imaging] [46-49,135-142]. [file 2045-5380-2-20-S1.doc]

Table 2 **Structural** **Neuroimaging** **Findings** **in** **Panic** **Disorder** **[Magnetic** **Resonance** **Imaging]**

| **Study** | **Subjects** | **No**. **of** **Subjects** **(Women**) | **Mean** **Age** (**SD**) | **Clinical** **State** | **Duration** **of** **Illness** (**Years**) | **Comorbid** **Depression** | **Comorbid** **Agoraphobia** | **Medication** **Status** | **Field** **Strength** (**Tesla**) | **Slice** **Thickness** (**mm**) | **Image** **Analysis** **Method** | **Adjustments** | **Amygdala** | **Hippocampus** | **Ventromedial** **Prefrontal** **Cortex** | **Other** **Brain** **Regions** |
| --- | --- | --- | --- | --- | --- | --- | --- | --- | --- | --- | --- | --- | --- | --- | --- | --- |
| **Studies** **Whose** **Results** **Indicate** **Amygdalar** **Involvement** | | | | | | | | | | | | | | | | |
| Asami, 2009 [23] | PD | 24 (15) | 36.3 (9.8) M 33.4(9.1) F 39.2(10.5) | PDSS 8.3 (4.6) | 3.9 (3.4) | History, n=3; Current dysthymia, n=1 | History, n=10; Current, n=3 | 19 patients receiving antidepressants (15 on SSRI, 2 on SNRI, 2 on TCA); 17 patients were receiving BDZ | 1.5 | 1.5 | Optimized VBM | Intracranial volume, self-SES, age , sex | Right amygdalar volume▼ *volume reduction was significantly greater in the males | No significant difference | Right Volume▼  *specific to female patients | Bilateral dorsomedial cortex, right anterior cingulate cortex, bilateral insular cortex, occipitotemporal gyrus and left cerebellar vermis volume▼ volume reduction in bilat. Insulrcortex was significantly greater in the males |
|  | HC | 24 (15) | 36.3 (8) M 33.2 (5.2) F 39.3 (10.8) |  |  |  |  |  |  |  |  |  |  |  |  |  |
| Hayano, 2009 [24] | PD | 27 (17) | 38.2 (9.9) | PDSS 8.8 (4.8) | 5.4 (6.4) | History, n=3 | Current, n=11 | 25 patients were receiving medications (SSRI, BDZ, SNRI, TCA were either used alone or in combination) | 1.5 | 1.5 | Amygdala and hippocampus manually traced |  | Bilateral amygdalar volume▼ | No significant difference | Not assessed | Not assessed |
|  | HC | 30 (21) | 35.3 (10.5) |  |  |  |  |  |  |  | Optimized VBM with small volume correction using the amygdalar mask | Intracranial content | Right amygdalar volume▼ |  |  |  |
| Massana, 2003a [25] | PD | 12 (6) | 35.3 (5.6) | Number of panic attacks during the last 4 weeks 12.3 (11.3); HAMA 27.4 (10.0) | >6 months, n=8; <6 months, n=4 | None | Some degree, n=10 | Free for 2 weeks None had ever been treated with antidepressants | 1.5 | 1.2 | Amygdala, hippocampus, and temporal lobe manually traced | - | Bilateral amygdalar volume▼ | No significant difference | Not assessed | Not assessed |
|  | HC | 12 (6) | 35.0 (5.9) | - | - | - | - |  |  |  |  |  |  |  |  |  |
| Uchida, 2003[26] | PD | 11 (8) | 36.9 (11.9) | Not mentioned | 8.0 (6.7) | History, n=5; Current dysthymia, n=1 | Current, n=6 | SSRI, n=3; TCA, n=3; BDZ, n=1; SSRI and BDZ, n=1 | 1.5 | 0.97 | Amygdala, hippocampus, temporal lobe, temporal pole manually traced | - | Trend-level significance for bilateral amygdalar volume ▼ | Trend-level significance for left hippocampal volume ▼ | Not assessed | Left temporal lobe volume▼ |
|  | HC | 11 (6) | 34.3 (10.2) |  |  |  |  |  |  |  |  |  |  |  |  |  |
| **Studies** **Whose** **Results** **Do** **Not** **Indicate** **Amygdalar** **Involvement** **or** **Those** **Which** **Did** **Not** **Assess** **Amygdalar** **Structures** | | | | | | | | | | | | | | | | |
| Fujiwara, 2011 | PD | 38 (25) | 38.7 (10.2) | PDSS 10.7 (5.9) | 5.0 (6.5) | Current, n=6 | History, n=22; Current, n=11 | All on medication (SSRI, BDZ, SNRI, TCA, TetraCA alone/in combination) | 1.5 | 1.5 | Midbrain manually traced | Sex and intracranial content (head size) | Not assessed | Not assessed | Not assessed | Dorsal midbrain volume▲ |
|  | HC | 38 (25) | 37.0 (10.2) |  |  |  |  |  |  |  |  |  |  |  |  |  |
| Kartalci, 2011 | PD | 26 (15) | 35.1 (10.7) | PAS 31.3 (6.4) | 10.0 (8.5) |  | Current, n=8 | SSRI, n=11; SSRI and BDZ, n=4 | 1.5 | 1.5 | Pituitary gland manually traced | Age and intracranial volume | Not assessed | Not assessed | Not assessed | Pituitary gland volume▼ |
|  | HC | 27 (15) | 33.7 (9.1) |  |  |  |  |  |  |  |  |  |  |  |  |  |
| Roppongi, 2010 [27] | PD | 28 (18) | 38.4 (9.8) | PDSS 9.0 (4.9) |  |  |  | 25 patients receiving antidepressant (average amitriptyline equivalent dose, 74.0 mg) and anti-anxiety (average diazepam equivalent dose, 6.6 mg) drugs | 1.5 | 1.5 | Sulcogyral pattern classification; optimized VBM with small volume correction using the OFC mask | Age, sex, and intracranial volume | Not assessed | Not assessed | Not assessed | Right posterior-medial OFC volume ▼ in patients with PD with absent or single posterior orbitofrontal sulcus compared to HC subjects with absent or single posterior orbitofrontal sulcus |
|  | HC | 28 (18) | 37.8 (9.8) |  |  | History, n=1; Current, n=1; Current dysthymia, n=1 | Current, n=15 |  |  |  |  |  |  |  |  |  |
| Asami, 2008 [28] | PD | 26 (16) | 37.7 (10.1) | PDSS 8.7 (4.9) | 3.8 (3.3) | History, n=4; Current dysthymia, n=1 | Current, n=3 | SSRI, n=17; SNRI, n=2; TCA, n=2;BDZ, n=19 | 1.5 | 1.5 | ACC manual tracing; optimized VBM with small volume correction using the ACC mask | Intracranial volume | No significant difference | No significant difference | No significant difference | Right anterior cingulate cortex volume▼ |
|  | HC | 26 (16) | 38.2 (9.7) |  |  |  |  |  |  |  |  |  |  |  |  |  |
| Uchida, 2008[29] | PD | 19 (16) | 37.1 (9.8) | GAF 65.5 (14.7) | 8.3 (6.0) | History, n=9; Current, n=3; Current dysthymia, n=2 | Current, n=14 | Antidepressant, n=10; BDZ, n=1; Antidepressant and BDZ, n=4 | 1.5 | 1.0 | VBM | - | No significant difference | No significant difference | No significant difference | Left insula, left superior temporal gyrus, midbrain, ponds volume▲; Right anterior cingulate cortex volume▼ |
|  | HC | 20 (16) | 36.5 (9.9) |  |  |  |  |  |  |  |  |  |  |  |  |  |
| Protopopescu, 2006 [30] | PD | 10 (6) | 35.5 (9.7) | PDSS Range 6-16 | Range 1–24 years | Yes, but exact number is not mentioned | Current, n=2 | Only 1 patient receiving medication | 3 | 1.5 | VBM | Age, sex, age-by-sex interaction, scanner, intracranial volume | No significant difference | Ventral hippocampal volume▲ | No significant difference | Midbrain, rostral pons, right occipital, right middle temporal volume▲; prefrontal cortex, left middle cingulate gyrus, right caudate, left fusiform gyrus volume▼ |
|  | HC | 23 (11) | 28.7 (7.5) |  |  |  |  |  |  |  |  |  |  |  |  |  |
| Yoo, 2005 [31] | PD | 18 (9) | 33.3 (7.1) | PDSS 8.3(5.7) | 3.6 (2.2) | None | None | None receiving medication at the time | 3 | 0.7 | VBM | Age and sex | No significant difference | No significant difference | No significant difference | Putamen, right precuneus gyrus, right inferior temporal gyrus, right inferior frontal gyrus, left superior temporal gyrus, left superior frontal gyrus volume▼ |
|  | HC | 18 (7) | 32.0 (5.8) | - | - | - | - |  |  |  |  |  |  |  |  |  |
| Massana, 2003b [32] | PD | 18 (11) | 36.8 (11.3) | Not mentioned | Not mentioned | None | Some degree, n=15 | Not mentioned | 1.5 | 1.2 | Non-optimzed VBM | - | No significant difference | No significant difference | No significant difference | Left parahippocampus volume▼ |
|  | HC | 18 (10) | 3.6 (8.8) |  |  |  |  |  |  |  |  |  |  |  |  |  |
| Vythilingam, 2000 [33] | PD | 13 (10) | 38.0 (11.0) | Not mentioned | Not mentioned | Current and past, n=1 | Current, n=9 | Not mentioned | 1.5 | 3.0 | Hippocampus and temporal lobe manually traced | Whole brain volume | Not assessed | No significant difference | Not assessed | Bilateral temporal lobes volume▼ |
|  | HC | 14 (8) | 39.9 (9.0) |  |  |  |  |  |  |  |  |  |  |  |  |  |
| Fontaine, 1990 [34] | PD | 31 (21) | 31.1 (−) | Not mentioned the MRI test was done when the panic attacks and phobic behaviors had significantly improved | Not mentioned | Not mentioned | Not mentioned | All had been treated with BDZ Clonazepam mean dose 3.2mg daily for 1–3 months all patients took an additional 2 mg of clonazepam in the hours before the MRI tests | 1.5 | Not mentioned | Global rating of the MR images |  | Not assessed | Not assessed | Not assessed | Right temporal volume▼ |
|  | HC | 20 (13) | 32.9 (−) |  |  |  |  |  |  |  |  |  |  |  |  |  |

Abbreviations: (−), data not avalable; *BDZ*, benzodiazepine; *CCK-4*, cholecystokinin tetrapeptide; *FA*, fractional anisotropy; *GAF*, Global Assessment of Functioning; *HAMA*; Hamilton Anxiety Rating Scale; *HC*, healthy control; *OFC*, orbitofrontal cortex; *PAS*, Panic and Agoraphobia Scale; *PD*, panic disorder; *PDSS*, Panic Disorder Severity Scale; *SD*, standard deviation; *SNRI*, serotonin-norepinephrine reuptake inhibitor; *SSRI*, selective serotonin reuptake inhibitor; *TCA*, tricyclic antidepressant; *VBM*, voxel-based morphometry.
