# Supplementary material for: The role of the amygdala in the pathophysiology of panic disorder: evidence from neuroimaging studies
Source: Biol Mood Anxiety Disord. 2012 Nov 20;2:20. doi: 10.1186/2045-5380-2-20 (PMC3598964; doi:10.1186/2045-5380-2-20)
Supplement: Additional file 2 — Functional Neuroimaging Findings in Panic Disorder [Positron Emission Tomography/Single Photon Emission Computed Tomography] [73-77,81,83-86,88,100-103,105,106,143-148]. [file 2045-5380-2-20-S2.doc]

**Table 6.** Functional Neuroimaging Findings in Panic Disorder [Positron Emission Tomography/Single Photon Emission Computed Tomography]

| **Study** | **Subjects** | **No**. **of** **Subjects** (**female**) | **Mean** **Age** (**SD**) | **Comorbid** **Agoraphobia** | **Medication** **Status** | **Imaging** **Method** | **Study** **Paradigm** | | **Amygdala** | **Hippocampus** | **Ventromedial** **Prefrontal** **Cortex** | **Other** **Brain** **Regions** |
| --- | --- | --- | --- | --- | --- | --- | --- | --- | --- | --- | --- | --- |
| **Studies** **Whose** **Results** **Indicate** **Amygdalar** **Involvement** | | | | | | | | | | | | |
| **Neutral** **State** | | | | | | | | | | | | |
| Sakai, 2005 [41] | PD | 12(9) | 29.8 (6.2) |  | Free for at least 2 weeks | FDG-PET | In resting state (without experiencing panic attacks during scans) | | Bilateral amygdala glucose uptake▲ | Bilateral hippocampus glucose uptake▲ | No significant difference | Bilateral thalamus, midbrain, caudal pons, medulla and cerebellum glucose update▲ |
|  | HC | 22(14) | 26.3 (6.8) |  |  |  |  |  |  |  |  |  |
| Malizia, 1998 [42] | PD | 7 (3) | 38.1 (18.4) |  | Off medication for at least 6 months. Never have had a prescription for benzodiazepines for anxiety. | [^11^C]Flumazenil-PET | Neutral state (Nobody experienced a panic attack either during the scanning session or during the preparation period). | | Bilateral amygdala/hippocampus benzodiazepine receptor binding▼ | | Bilateral medial frontal, bilateral orbitofrontal, and anterior cingulate regional benzodiazepine receptor binding▼ | Bilateral thalamus, bilateral basal ganglia, bilateral lateral temporal, bilateral medial/lateral occipital, bilateral anterior/dorsolateral prefrontal, and right insula regional benzodiazepine receptor binding▼ |
|  | HC | 8 (0) | 38.9 (9.0) |  |  |  |  |  |  |  |  |  |
| Kaschka, 1995 [43] | PD with dysthymia or mild depression | 9 (4) | 30.2 (9.8) |  | TCA for at least 4 weeks with steady-state serum levels within a range of 150-200 ng/ml. | Iomazenil-SPECT | Resting state | | Left medial inferior temporal regional activity index▼ | | | Bilateral inferior temporal, and bilateral inferior frontal regional activity index▼ |
|  | Patients with a history of dysthymia | 9 (4) | 29.0 (8.5) |  | TCA with steady-state serum levels within a range of 150-200 ng/ml. |  |  |  |  |  |  |  |
| **Symptom** **Provocation** | | | | | | | | | | | | |
| Boshuisen, 2002 [44] | PD | 17(12) | 18 or older |  | Free for at least 3 weeks | [^15^O]H_2_O PET | H_2_^15^O PET scan, before and after a pentagastrin challenge | At rest (after the pentagastrin challenge) | Anticipatory anxiety: Significantly deactivated on the right side |  | Anticipatory anxiety: Orbitofrontal cortex, and the anterior cingulate gyrus▲ | Anticipatory anxiety: Left precentral gyrus, left insula ctivity▼ Anticipatory anxiety: The left parahippocampus, the superior temporal gyrus, the hypothalamus, and the midbrain activity▲ |
|  |  |  |  |  |  |  |  | Before the pentagastrin challenge |  |  | Anterior cingulate cortex▼ | The parahippocampus, the precentral gyrus, the inferior frontal gyrus activity and the anterior insula▼ The superior temporal lobe and the midbrain▼- |
|  | HC | 21(12) |  |  |  |  |  |  |  |  |  |  |
| Javanmard, 1999 [45] | Right handed, naive to CCK-4 injection HC | 20(10) | 30 |  | Any individuals who used any psychoactive medication within a week prior to the scanning were excluded | [^15^O]H_2_O PET | Early scan or late scan covering the first or the second minute after CCK-4 bolus injection, respectively | Early scan |  |  | Early scan: Medial frontal region▲ Anticipatory anxiety: Anterior cingulate region rCBF▲ | Hypothalamic region rCBF▲ Occipital regions rCBF▼ |
|  |  |  |  |  |  |  |  | Late scan | Right amygdala rCBF▲ |  |  | Claustrum-insular region rCBF▲ Left superior temporal rCBF▲ Cerebellar rCBFd▲ |
| Servan-Schreiber, 1998 [46] | HC | 10(5) | 20.4 (3.66) |  |  | [^15^O]H_2_O PET | Limbic stimulation with intravenous procaine | Procaine | Bilateral amygdala rCBF▲ |  | Anterior cingulate gyrus activation▲ | Bilateral parahippocampal gyri, left insular cortex rCBF▲ Left inferior parietal lobule, right thalamus, bilateral cerebellum rCBF and right pulvinar▼ |
|  |  |  |  |  |  |  |  | Placebo |  |  |  |  |
| Benkelfat, 1995 [47] | HC | 8(3) | Male 26.4 (4.0) / |  | Free for 1 week | MRI [^15^O]H2O PET | CCK-4 | | Bilateral claustrum-insular-amygdalar region CBF▲ |  | Anticipatory anxiety condition: Left orbitofrontal cortical rCBF▲ CCK-4 condition: Left anterior cingulate▲ | Anticipatory anxiety condition: Cerebellum rCBF▲ rCBF in the bilateral region adjacent to the frontotemporal poles▲ |
|  |  |  |  |  |  |  | Placebo (with or without anticipation) | |  |  |  |  |
|  |  |  | Female 23.7 (1.5) |  |  |  |  |  |  |  |  |  |
| **Treatment**-**Related** | | | | | | | | | | | | |
| Sim, 2010 [48] | PD | 5 | 42.6 (7.0) |  | Paroxetine (12.5-37.5 mg/day) for 12 weeks | FDG-PET | 12 weeks of paroxetine trial | | Right amygdala glucose metabolism▲ after treatment | No significant difference | No significant difference | Bilateral precentral gyrus, bilateral middle frontal gyrus, right caudate body, right putamen, left insula, left parahippocampal gyrus, left inferior frontal gyrus glucose metabolism▲ after treatment |
| Nash, 2008 [49] | Untreated PD | 9 |  |  | Medication-free for 3 months | 5-HT_1A_-PET | measure receptor binding in patients PD in the untreated state and after recovery on treatment with SSRIs | | Untreated vs. control: Binding potential in the amygdala▼ | Treated vs. control: Binding potential in the hippocampus▼ | Treated vs. untreated: Binding potential in the orbitofrontal cortex▼ | Untreated vs. control: Binding potential in the raphe and anterior lateral temporal cortex▼ Treated vs. control: Binding potential in the raphe and anterior medial temporal cortex▼ |
|  | Treated PD with SSRI | 7 |  |  | Open-label treatment with paroxetine hydrochloride or sertraline (1 patient: daily dose 50 mg, duration of treatment 24 months) |  |  |  |  |  |  |  |
|  | HC | 19 |  |  |  |  |  |  |  |  |  |  |
| **Studies** **Whose** **Results** **Do** **Not** **Indicate** **Amygdalar** **Involvement** **or** **Those** **Which** **Did** **Not** **Assess** **Amygdalar** **Regions** | | | | | | | | | | | | |
| **Spontaneous** **Panic** **Attack** | | | | | | | | | | | | |
| Fischer, 1998 [50] | Healthy volunteer | 1(1) | 42 | None | None | [^15^O]H2O PET | Spontaneous panic attack during fear conditioning with electric shocks and visual white noise | | No significant difference | No significant difference | Right posterior orbitofrontal rCBF▼  Right anterior cingulate rCBF▼  Right prelimbic rCBF▼ | Right temporal cortical rCBF▼ |
|  | Healthy volunteer | 5(5) | 24(2.5) |  |  |  |  |  |  |  |  |  |
| **Neutral** **State** | | | | | | | | | | | | |
| Eren, 2003 [51] | PD | 22(20) | 41.6 (11.5) | n = 9 |  | 99mTc-HMPAO -SPECT | In neutral state | | No significant difference of the left/right rCBF ratios in the medial temporal regions | No significant difference of the left/right rCBF ratios in the medial temporal regions | ▲ Left/right rCBF ratios in the medial frontal region. (Not significant after multiple comparison correction.) | ▼Left/right rCBF ratios in the inferior frontal cortex. |
|  | HC | 19(16) | 35.8 (10.7) |  |  |  |  |  |  |  |  |  |
| Bisaga, 1998 [52] | PD | (6) | 44.5 (4.2) |  | Free for 1 month | FDG-PET | In neutral state (Lactate infusion was begun after completion of the scan) | | No significant difference | Left hippocampus glucose metabolism ▲ | No significant difference | Left parahippocampal area glucose metabolism▲ Right inferior parietal, right superior temporal brain regions glucose metabolism▼ |
|  | HC | (6) | 39.8 (9.3) |  |  |  |  |  |  |  |  |  |
| De Cristofaro, 1993 [53] | PD | 7 (5) | 24.5 (2.5) | n = 2 had panic disorder without agoraphobia n = 5 suffered from panic disorder with phobic avoidance. | Free for at least 2 weeks | 99mTc-HMPAO -SPECT | In neutral state | | Due to limited resolution of the images, hippocampal regions may include amygdalar regions | Bilateral hippocampal regions blood flow▼ | No significant difference | Inferior frontal cortex rCMRglu asymmetry index▲ Left occipital cortex blood flow▲ |
|  | HC | 5 (3) | 27.8 (6.9) |  |  |  |  |  |  |  |  |  |
| Nordahl, 1990 [54] | PD | 12 (7) | 35.5 (11.4) |  | n = 1 drug-naive n = 1 free for at least 1 year n = 10 free for 20.3 (SD 10.19) days | FDG-PET | Auditory discrimination task | | Not assessed | Left/right hippocampal ratio of rCMRglu▼ | Left anterior cingulate region metabolic rates▼ Left medial orbital frontal region metabolic rates▲  Right prefrontal region metabolic rates▲ | Left inferior parietal lobe metabolic rates▼ |
|  | HC | 30 (14) | 33.3 (9.8) |  |  |  |  |  |  |  |  |  |
| Reiman, 1986 [55] | PD | 16 (11) | 34 (Range 22–52) | None had extensive phobic avoidance (i.e., agoraphobia) | n = 4 were receiving tricyclic antidepressants and alprazolam. | [^15^O]H_2_O, [^15^O]CO, and [^15^O]O_2_ PET | In neutral state | | Not assessed | Not assessed | Not assessed | Left/right ratios of parahippocampal rCBF , whole brain metabolism▼ in lactate-induced panic-vulnerable PD patients relative to HC. |
|  | HC | 2 5(9) | 35 (Range 19–74) |  |  |  |  |  |  |  |  |  |
| Reiman, 1984 [56] | PD | 10 |  |  |  | [^15^O]H2O PET | In neutral state | | No significant difference | No significant difference | No significant difference | Left/right ratios of parahippocampal rCBF▼in patients who had a positive response to lactate infusion compared with those without and HC. |
|  | HC | 6 |  |  |  |  |  |  |  |  |  |  |
| **Symptom** **Provocation** | | | | | | | | | | | | |
| Kent, 2005 [57] | Untreated PD | 5 (4) / One female subject excluded later | 27.2 (3.2) |  | None | [^15^O]H2O PET | Doxapram intravenous injection | | No significant difference | No significant difference | No significant difference / Baseline orbitofrontal rCBF predicted anxiety scores in response to doxapram injection |  |
|  | HC | 5 (3) | 31.6 (6.8) |  |  |  |  |  |  |  |  |  |
| Reiman, 1989 [58] | PD Group 1 (Positive lactate-induced anxiety attack) | 8 (5) | 28 (Range 22–35) |  | n = 1 taking 150 mg/d of oral imipramine hydrochloride All other patients medication free for at least 2 weeks | [^15^O]H2O PET | Sodium lactate infusion | | No significant difference | No significant difference | No significant difference | ▲Blood flow increase in bilateral temporal poles, bilateral insular cortex, claustrum, lateral putamen, near the superior colliculus, or the left anterior cerebellar vermis in patients who panicked compared with those who did not and HC. |
|  | PD Group 2 (Negative lactate-induced anxiety attack) | 9 (5) | 38 (Range 28–52) |  |  |  |  |  |  |  |  |  |
|  | HC | 15 (8) | 25 (Range 22–30) |  |  |  |  |  |  |  |  |  |
| Stewart, 1988 [59] | PD | 10 | Patients who panicked 34.3 (11.3) / Patients who did not panicked 34.2 (7.4) |  | All tricyclic antidepressant, MAOI, or alprazolam-naive, n = 7 medication-free, n = 2 oxazepam, n = 1 clorazepate | Xenon-133 SEPCT | Sodium lactate infusion | | Not assessed | Not assessed | Not assessed | Total left hemispheric blood flow▼ in patients who panicked than those who did not and HC. ▲Bilateral occipital regional blood flow increase in patients who panicked that those who did not and HC. |
|  | HC | 5 | 31.6 (4.8) |  |  |  |  |  |  |  |  |  |
| Woods, 1988 [60] | PD | 6 (4) | 30(3) |  |  | 99mTc-HMPAO -SPECT | Yohimbine infusion | | Not assessed | Not assessed | Not assessed | ▼Bilateral frontal blood flow decrease by yohimbine infusion relative to placebo infusion. |
|  | HC | 6 (4) | 30(10) |  |  |  |  |  |  |  |  |  |
| **Treatment**-**Related** | | | | | | | | | | | | |
| Sakai, 2006 [61] | PD | 12(9) | 29.8 (6.2) |  | Fluoxetine or cognitive behavioral therapy-naive and medication free for 2 weeks prior to study enrollment. | FDG-PET | Before and after 10 sessions of cognitive-behavioral therapy for 6 months | | No significant difference | Right hippocampus glucose utilization▼ | Bilateral medial prefrontal cortex glucose utilization▲ Left anterior cingulate glucose utilization▼ | Left cerebellum, and pons glucose utilization▼ |
| Prasko, 2004 [62] | PD assigned to antidepressant treatment | 6 |  |  | CBT or antidepressants for 3 months | FDG-PET | Before and after treatment for 3 months | | No significant difference | No significant difference | Right medial frontal▼ Left medial frontal▲ | Right superior/middle/inferior frontal▼ Right superior/middle/transverse temporal▼ Left middle frontal▲ Left superior/middle/transverse temporal▲ |
|  |  |  |  |  |  |  |  |  |  |  |  | Right superior/inferior frontal▼ Right inferior temporal▼ Left inferior frontal▲ Left middle temporal▲ Insula▲ |
|  | PD assigned to CBT | 6 |  |  |  |  |  |  |  |  |  |  |
| Nordahl, 1998 [63] | Imipramine-treated PD | 9(4) | 33.3 (7.2) |  | 6 weeks of treatment with imipramine, including 2 weeks at their final dose with a range of 50–150 mg | FDG-PET | Continuous auditory performance task to give a standardized condition | | Not assessed | Abnormally low L/R hippocampal | Posterior orbital frontal rCMRglc▼ | Left/right posterior inferior prefrontal rCMRglc ratio▼ |
|  | Untreated PD | 12(7) | 35.5(11.4 |  | 1 had never been on medication 1 had been of of medications for more than 1 year n = 10 the ange of time off medications was 20.3_ + 10.19 days |  |  |  |  |  |  |  |
|  | HC | 43(22) | 30.2(9.2) |  |  |  |  |  |  |  |  |  |

Abbreviations : *CBT*, cognitive behavioral therapy; *CCK-4*, cholecystokinin tetrapeptide; *FDG*, 18 F-fluorodeoxyglucose; *HC*, healthy control; *MAOI*, monoamine oxidase inhibitor; *MDD*, major depressive disorder; *PD*, panic disorder; *rCBF*, regional cerebral blood flow; *SD*, standard deviation; *SPECT*, single photon emission computed tomography; *SSRI*, selective serotonin reuptake inhibitors; *99mTc-HMPAO*, 99mTc-hexamethylpropyleneamineoxime.

*Results from these studies [55,56,58] were subsequently retracted.
