# Supplementary material for: The role of the amygdala in the pathophysiology of panic disorder: evidence from neuroimaging studies
Source: Biol Mood Anxiety Disord. 2012 Nov 20;2:20. doi: 10.1186/2045-5380-2-20 (PMC3598964; doi:10.1186/2045-5380-2-20)
Supplement: Additional file 3 — Functional Neuroimaging Findings in Panic Disorder [Functional Magnetic Resonance Imaging] [78-80,82,87,89-91,93,94,97-99,149-153]. [file 2045-5380-2-20-S3.doc]

**Table 7.** Functional Neuroimaging Findings in Panic Disorder [Functional Magnetic Resonance Imaging]

| **Study** | **Subjects** | **No**. **of** **Subjects** (**Female**) | **Mean** **Age** (**SD**) | **Clinical** **State** | **Comorbid** **Depression** | **Comorbid** **Agoraphobia** | **Medication** **Status** | **Field** **Strength** (**Tesla**) | | **Slice** **Thick**-**ness** (**mm**) | **Study** **Paradigm** | | | **Amygdala** | **Hippocampus** | **Ventromedial** **Prefrontal** **Cortex** | **Other** **Brain** **Regions** |
| --- | --- | --- | --- | --- | --- | --- | --- | --- | --- | --- | --- | --- | --- | --- | --- | --- | --- |
| **Studies Who Results Indicate Amygdalar Involvement** | | | | | | | | | | | | | | | | | |
| **Spontaneous Panic Attack** | | | | | | | | | | | | | | | | | |
| Dresler, 2011 [64] | Specific phobia, PD | 2 (0) | 28 and 31 |  | n=1 |  | metropolol olanzapine+ lorazepam+mirtazapine | 1.5 | | 4 | Spontaneous panic attack | | | Right▲ | Insula▲ |  |  |
| Spiegelhalder, 2009 [65] | Restless leg syndrome | 1 (1) Case report | 46 | No history of PD, claustrophobia or psychiatric disorders |  |  |  | 1.5 | | 5 | Spontaneous panic attack  - | | | Positive correlation of left amygdala with heart rate |  |  | Negative correlation of left middle temporal gyrus with heart rate |
| Pfleiderer, 2007 [66] | PD | 1 (1) Case report | 26 | 8 year-history of PD |  | Current | SSRI | 3.0 | | 3.6 | Spontaneous panic attack  - | | | Right▲ | - | ACC▲ | Auditory cortex▲ Right lateral globus pallidus▲ Middle temporal gyrus▲ Thalamus▲ Medial and inferior frontal gyrus▲ Right putamen▲ |
| Symptom Provocation | | | | | | | | | | | | | | | | | |
| Eser, 2009 [67] | HC | 16(0) | 25.6 (4.2) |  |  |  | Free for 4 weeks | 1.5 | | 3 | CCK-4 injection | | | ▲Number of activated voxels in the amygdala ROI |  | ▲(ACC ventral part) | Middle and superior frontal gyrus▲ Precuneus▲ Middle and superior temporal gyrus▲ Occipital lobe▲ Sublobar areas▲ Cerebellum▲ Brainstem▲ |
| Activation Probe | | | | | | | | | | | | | | | | | |
| Tuescher, 2011 [68] | PD | 8 (4) | 37 (Range24-50) |  | Past n=1, Curret n=1 | Current, n=1 | SSRI and bupropion, n=1 | 3.0 | 5 | | Cognitively instructed fear conditioning in Threat or Safe condition | | | In the Threat vs Safe condition: Extended amygdala▼ in PD patients compared to PTSD patients | No significant difference | In the Threat vs Safe condition: Subgenual cingulate▼ in PD patients compared to PTSD patients. | In the Threat vs Safe condition: Ventral striatum▼ in PD patients compared to PTSD patients. |
|  | PTSD | 8 (4) | 42 (Range 37–50) |  | Current n=2, past n=1 | None | None |  |  |  |  |  |  |  |  |  |  |
|  | HC | 8 (4) | 35 (Range24-49) |  |  |  |  |  |  |  |  |  |  |  |  |  |  |
| Wittmann, 2011 | PD | 16(8) | Male 36.4 (14.5) Female 37.9(14.3) | Male HAM-A 24.6(7.6) CGI 5.3(0.46) Female HAM-A 23.8(4.0) CGI 5.3(0.89) |  | Current, n=16 | Free of psychopharmacological treatment for at least 4 weeks | 3 | | 3.8 | Westphal-Paradigm (with agoraphobia-specific stimuli) | | | Left▲ during cued compared to uncued agoraphobia-related pictures |  |  | PPA▲ Right insula▲ Precuneus▲ Middle temporal gyrus/angular gyrus▲ |
| Ohrmann, 2010 [69] | PD women | 12 | 38.2 (9.2) | STAI trait anxiety 50.3 (13.4) | n=2 |  | n=7 citalopram | 3.0 | | 3.5 | Facial xstimuli of fearful, angry, happy, and neutral expressions of 10 individuals. Comparison of activation (each face stimulus vs. no-face stimulus) between women patients and men patients. (Expression: women increased▲, women decreased▼) | | angry | Bilateral amygdala▲ | No significant difference | No significant difference | Middle frontal▲ Inferior frontal▲ Insula▲ Superior/middle/inferior temporal▲ Insula▲ Fusiform▲ Caudate, putamen, and thalamus▲ Caudate, precuneus▼ |
|  | PD men | 8 | 34.6 (9.1) | STAI trait anxiety 46.5 (12.1) | n=1 |  | n=3 citalopram |  |  |  |  |  | Fearful | Bilateral amygdala▲ | No significant difference | Middle cingulate▲ | Insula▲ Middle occipital▲ Lingual▲ Precentral▲ Caudate▲ |
|  |  |  |  |  |  |  |  |  |  |  |  |  | Neutral | Bilateral amygdala▲ | No significant difference | Anterior/middle/posterior cingulate▲ | Putamen, thalamus, caudate, fusiform▲ Lingual▲ |
|  |  |  |  |  |  |  |  |  |  |  |  |  | Happy | Bilateral amygdala▲ | No significant difference | No significant difference | Lingual▲ Caudate▲ Postcentral▲ Superior/middle temporal▲ Insula▲ Cerebellar vermis▲ |
| Beutel, 2010 [70] |  |  |  |  |  |  |  | 1.5 | | 5 | Emotional/linguistic go/nogo trials with affective words (Negative/Positive/ Neutral), PD patients scanned before (S1)/after (S2) a short-term psychodynamic inpatient treatment | | | [S1] Inhibition-related activation in neutral context: Left▲ Inhibition-related activation in positive context: Right▲ | [S1] Negative: Left▲ [S2] Neutral: Left▲ |  | [S1] Negative/Positive: SMA▲ Negative/Positive: LPFC▼ (Negative: VLPFC; Positive: DLPFC) [S2] Negative: Left middle temporal gyrus▲ |
|  | HC | 18(9) | 29 (Range 21–40) |  |  |  | Migraine prophylaxis, n=1 |  |  |  |  |  |  |  |  |  |  |
| Pfleiderer, 2010 [71] | PD | 20(10) | 31.45 (12.4) |  | Secondary lifetime diagnoses n=5 | Secondary lifetime diagnoses, n=7 | SSRI and/or TCA, n=9; NRI, n=1 | 3.0 | | 3.6 | 3 Auditory stimulation cycles | 5-HTTLPR | PD SS/SL, n=14 | Interaction of left amygdala activation and the 5-HTTLPR S allele. For right amygdala, there was a trend level significance for the interaction. |  | ACC▲ | Right middle frontal gyrus▲ Right inferior frontal gyrus▲ Left inferior temporal gyrus▲ Right insula▲ Left parahippocampal gyrus▲ |
|  | HC | 20(10) | 31.85 (11.3) |  |  |  |  |  |  |  |  |  | PD LL, n=6 |  |  |  |  |
|  |  |  |  |  |  |  |  |  |  |  |  |  | HC SS/SL, n=11 |  |  |  |  |
|  |  |  |  |  |  |  |  |  |  |  |  |  | HC-LL, n=9 |  |  |  |  |
| Chechko, 2009 [72] | PD (Remitted state) | 18 (10) | 34.4 (8.0) | PAS 3.3 (1.7) |  | n=14 | All receiving monotherapy with an SSRI | 1.5 | | 4 | Emotional conflict paradigm | Preceded by congruent trials | | No significant difference | No significant difference | Dorsal ACC, dorsomedial prefrontal cortex▲ | Right middle and superior frontal gyrus▲ Right supramarginal, superior and inferior parietal gyrus▲ Bilateral precuneus▲ |
|  | HC | 18 (10) | 30.0 (6.4) |  |  |  |  |  |  |  |  | Preceded by incongruent trials | | Right temporomesial area (including amygdala)▲ | No significant difference | No significant difference | Left parahippocampal gyrus▲  Right middle and superior temporal gyrus▲ Bilateral brainstem▲ |
| Domschke, 2008 [73]  ) | PD | 20(12) | 36.75 (9.39) |  |  | Secondary lifetime diagnoses, n=5 | SSRI, n=10 | 3 | | 3.5 | Visual presentation of emotional faces | COMT val158met | AA, n=7 |  |  |  |  |
|  |  |  |  |  |  |  |  |  |  |  |  |  | AG/GG, n=13 | Fearful: Right▲ in patients carrying at least one 158val allele |  | Angry: Left, less deactivation Happy: Less deactivation Fearful: Left OFC |  |
| Pillay, 2007 [74] | PD | 8(4) | 36 (8.3) | HAM-A 9.6 (3.1) HAM-D 5.1 (2.4) STAI-Y1 43.9 (3.5) STAI-Y2 46.6 (5.6) | At least one lifetime episode, but free of MDD at least 5 years |  | BDZ, n=2; SSRI and BDZ, n=5; SSRI, BDZ and gabapentine, n=1 | 1.5 | | 6 | Affect perception task (Happy/Neutral Faces) | | | Neutral: Left▲ |  | Happy:▲ Neutral:▲ |  |
|  | HC | 8(4) | 25.8 (3.5) |  |  |  |  |  |  |  |  |  |  |  |  |  |  |
| Pillay, 2006 [75] | PD | 8(4) | 36 (8.3) | HAM-A 9.6 (3.1) HAM-D 5.1 (2.4) STAI-Y1 43.9 (3.5) STAI-Y2 46.6 (5.6) | At least one lifetime episode of MDD, but had been free of MDD at least 5 years prior to the study |  | BDZ, n=2; SSRI and BDZ, n=5; SSRI, BDZ and gabapentine, n=1 | 1.5 | | 6 | Affective faces (Fearful/ Neutral) | | | Fearful: Bilateral▼ (Greater number of voxels were deactivated in right amygdala than in left amygdala) |  | Fearful:▼ Neutral:▲ |  |
|  | HC | 8(4) | 31.6 (8.8) |  |  |  |  |  |  |  |  | | |  |  |  |  |
| Domschke, 2006 [76] | PD | 20(12) | 36.75 (9.39) |  | Past, n=5 |  | SSRI, n=10 | 3.0 | | 3.5 | Presentation of affective faces (fearful/angry/ happy/neutral) to genotyped patients | 5HT_1A_ -1019C/G | GG, n=5 | Happy: Left ▲ |  | Fearful: Right▼ | Fearful: Right OFC▼ |
|  |  |  |  |  |  |  |  |  |  |  |  | 5-HTTLPR | CC/CG, n=15 |  |  |  |  |
|  |  |  |  |  |  |  |  |  |  |  |  |  | SS/SL, n=13 | Happy: ▲ |  |  |  |
|  |  |  |  |  |  |  |  |  |  |  |  |  | LL, n=7 |  |  |  |  |
| van den Heuvel, 2005 [77] | PD | 15(7) | 33.7 (2.5) | BSQ 38.1 (3.3) |  |  | Free for 4 weeks | 1.5 | | 3.6 | Cognitive/emotional Stroop task | | | Right▲ | Right▲ | ▲ | Anterior PFC▲, Inferior parietal cortex▲, Right DLPFC▲, VLPFC▲, OFC▲, Thalamus▲, Middle temporal cortex▲ |
|  | HC | 19(19) | 30.3 (1.9) |  |  |  |  |  |  |  |  |  |  |  |  |  |  |
| Studies Whose Results Do Not Indicate Amygdalar Involvement or Those Which Did Not Assess Amygdalar Regions | | | | | | | | | | | | | | | | | |
| Symptom Provocation | | | | | | | | | | | | | | | | | |
| Schunck, 2006 [78] | PD | 12(0) | 27 (4) |  |  |  |  |  | |  |  |  |  |  |  |  |  |
|  | HC | 8(5) | 35 |  |  |  | Free for at least 2 weeks | 3.0 | | 4 | CCK-4 injection | | | No significant difference | -No significant difference | CCK-4 injection: Anterior cingulate cortex▲ Posterior cingulate▲ | CCK-4 injection: Cerebellar vermis▲ Insula▲ Temporal poles▲ |
| **Activation** **Probes** | | | | | | | | | | | | | | | | | |
| Marchand, 2009 [79] | PD | 12(12) | 27.5 (4.9) |  |  |  | BDZ, n=2; SSRI, n=1; SSRI and BDZ, n=3 | 3.0 | | 3 | Complex motor activation paradigm for the non-dominant hand | | | No significant difference | No significant difference | No significant difference | Putamen▼ Right temporal/occipital region▼ |
|  | HC | 18(18) | 26.4 (3.9) |  |  |  |  |  |  |  |  |  |  |  |  |  |  |
| Maddock, 2003 [80] | PD | 6(4) | 34 |  | None | n=5 | All had not taken medications affecting psychiatric or cerebrovascular function for ≥14 days | 1.5 | | 6 | Valence judgment during threat-related and neutral words presentation | | | No significant difference | No significant difference | Posterior cingulate▲ | Left middle frontal▲ |
|  | HC | 8(5) | 35 |  |  |  |  |  |  |  |  |  |  |  |  |  |  |
| Bystritsky, 2001 [81] | PD | 6(3) | 31.8 |  |  |  | Free for 2 weeks | 3.0 | | 4 | Directed imagery of neutral, moderate and high anxiety situations | | | No significant difference | ▲ | Bilateral anterior cingulate, posterior cingulate, and orbitofrontal cortex▲ | Right inferior frontal▲ |
|  | HC | 6(3) | 31.8 |  |  |  |  |  |  |  |  |  |  |  |  |  |  |

Abbreviations: *BDZ*, benzodiazepine; *BSQ*, Body Sensations Questionnaire; *CCK-4*, cholecystokinin tetrapeptide; *CGI*, Clinical Global Impression; *DLPFC*, dorsolateral prefrontal cortex; *GAD*, generalized anxiety disorder; *HAM-A*, Hamilton Anxiety Rating Scale; *HAM-D*, Hamilton Depression Rating Scale; *HC*, healthy control; *LPFC*, lateral prefrontal cortex; *NRI*, norepinephrine reuptake inhibitor; *OFC*, orbitofrontal cortex; *PD*, panic disorder; *PFC*, prefrontal cortex; *PPA*, parahippocampal place area; *PTSD*, post-traumatic stress disorder; *ROI*, region of interest; *SD*, standard deviation; *SMA*, supplementary motor area; *SNRI*, serotonin-norepinephrine reuptake inhibitor; *SSRI*, selective serotonin reuptake inhibitor; *STAI*, State Trait Anxiety Index; *TCA*, tricyclic antidepressant; *VLPFC*, ventrolateral prefrontal cortex.
